# Book Reviews

**Published:** 1875-06-15

**Authors:** 


### Book Reviews.

REST IN NERVOUS DISEASE — ITS USE AND ABUSE. By S. Weir Mitchell, M.D. 20 pp. Price, 30c.

This is No. 4 of the series of American Clinical Lectures now being issued in monthly parts by G. P. Putnam's Sons.

A few extracts will show the tenor of the essay:

"Rest and unrest," the author says, "have had their days and fashions in medicine; but be you sure that he who can tell when the one is wanted, and when the other, is a man who is a master in the ways of healing. Surgeons and doctors for a long while have been using rest as one means of curing disease. Not by any means all of them have distinct views as to what it is they do when they put at rest a limb or the whole body; and yet this is what we most want to know. Unhappily, we lack as yet some of the factors needed to work out this hard equation; and until these are given we must in part only guess at the physiological results of rest, for to-day no man can tell me fully what is the difference in the products of the life of a limb at positive rest and in active motion. In fact, most that we know on this matter is purely empirical, and is in the shape of coarse clinical results. Still, even these teach certain things which you will do well to bear in mind. First of all is the thought, which should be ever with us, that few medical means are without their evil side. In our efforts to help, we too often harm, and we must take prudent care always that in causing the largest share of good we give rise to the least amount of ill. The one goes with the other as surely as shadow with light. To no medical measure does this caution more apply than to the use of rest.

"When you put patients in bed, and forbid them to rise or to make use of their muscles, you at once lessen appetite, weaken, in many cases, digestion, constipate the bowels, and enfeeble circulation. To say how all this arises, would need not a lecture, but a book, and I can only hint at what I might call headings for thought. Defect of circulation is the main business to think about. A man in bed has his heart-beats brought down in number and also in force. Then there is for him no longer the constant momentary pumping out of blood from active muscles, and these aids to the heart failing, the distant local circulations suffer, and the blood flows around the muscles and not through them, and the skin ceases to be flushed by exercise and becomes pale and shrunken. To be small one moment and large the next is a condition of health for the vessels, and this fails for the want of exercise, so that when a man lies in bed the muscles lose tone, and when he gets up of a sudden this is seen in the way the blood column enlarges the lower vessels, and, leaving the head, causes faintness. Of these well-known facts I remind you only that you may the more fully see why I dwell so much on the means which must be used with rest in order to take from its avoidable evils.

"Think, then, when you put a person in bed that you are lessening the heart-beats some twenty a minute, nearly a third; that you are making the tardy blood to linger in the by-ways of the blood-round, for it has its by-ways; that rest prone, binds the bowels, and tends to destroy the desire to eat; and that muscles in rest too long get to be unhealthy and shrunken in substance. Bear these ills in mind, and be ready to meet them, and you will have answered the hard question of how to help by rest without hurt to the patient.

"But what is it that rest does for your sick man? We have seen, in brief, how it hurts; how does it help? When first I came to ask myself this question, I found that though in many cases I was sure of its usefulness, I was by no means sure why it gave rise to such helpful results; nor am I now much better off in this matter, as concerns some of the diseases in which I am most secure of aid from it."

Rest in neuralgias is first considered, and a variety of cases given.

"I once treated a case of infra-maxillary neural pain by rest. I forbade laughing and talking, and gave only fluid food. These means made less the number of fits of pain, which were usually about fifty a day. On the first day of rest they became thirty-three, and in four days came down to eleven, but these were very bad, as if with lessening the number we had made greater the pain. I found in this case that rest only helped but did not cure, nor is it ever much more than an aid to other means. Could we have put a stop to swallowing, no doubt we should have done still better, for the more she swallowed the worse grew the pain. Motion of the part, then, increases nerve pain, and motion or active function of parts far from it may do the same. No one can yet say why this is as regards the fifth nerve. The motions we speak of do not all of them mechanically disturb the nerve. In many cases they cannot. No doubt there are close relations in the sensorium between the centres which get impressions of all kinds from face, and throat, and mouth, and stomach, and when one little centre becomes irritable from disease, or the steady acting of an outside cause of disorder, like a pinched nerve, it soon begins to feel morbidly, through other ganglia, the normal impressions which every functional act brings to them, and through them to it.

"Now this is not an explanation; it is only a way of stating the facts, but it is a way which perhaps may aid you to see them in a clearer light. Suppress, by rest, the number of normal excitations which reach the over-sensitive nerve cells, and you suppress some of the causes of pain, because an excitable centre feels all things as pain, and grows more and more sensitive, like a man in anger whose wrath is fed by everything.

"The use of these facts gets to be plainer in neuralgias of the limbs. Some of you will recall a case of neuralgia from a wound of the median nerve-branches in the hand, which you saw last year. The irritations lasted long, and were so constant that the whole of the sensory centres belonging to the arm got at last to be distressingly alive to outside impressions, so that the faint messages sent to the brain from the motions of distant muscles in the forearm and arm were all felt in the centres as pain.

"It has long been my practice to insist that patients in the early stages of spinal congestion, meningitis, and chronic myelitis, especially in the very well localized forms the latter disease may assume, should lie down whenever they are indoors. If I can carry my point, I like very much to put these cases in bed, and at perfect rest for a few weeks, and my reasons for doing so are these: Erect posture, and walking heavily, tax the spinal ganglia, and never more so than when every step is labor. The prone position puts them at rest. It is easiest to repair when the machine is not in full action, and so rest is to be wished for in a disordered spine. Besides which I could tell you case on case of these troubles, in which some sudden fatigue has brought about an abrupt increase in the gravity of the disease, while I have never seen ill come of rest, even though it caused no gain.

"In cases of chronic meningitis, such as I have seen come of injury, or from the downward passage of cerebral meningitis, after sunstroke, rest, with counter-irritants, is almost the only very good means; rest, long and complete, is the one utmost want. Nor need I say much as to the treatment by pure rest of the meningomyelitis of Pott's disease, in which you have all seen the most perfect success in this house from the use of the prone position without the classical treatment by the hot iron, which is certainly sometimes useful, but in many cases needless, despite Charcot's recent and most authoritative statement.

"I come last in my hasty review to a class of cases in which the use of rest is entirely my own idea, and in which I am sure I have gotten results of great value — results which happily you have seen with me in this very hospital. I allude to the use of rest in locomotor ataxia, in its early stages of pain chiefly and most surely, but also in any case of posterior sclerosis before it has gone too far. You will ask me what I mean by this phrase, "gone too far;" and I answer that it is vague, because I really am, as yet, unable to set the limit beyond which rest is useless. I have seen it strangely relieve old cases of ataxia in which there was pain, and with the relief of pain there was also unhoped-for relief of other symptoms.

"I have tried in this lecture to make clear my views as to the evil and the good of rest, but I have failed to satisfy myself if I have not set in strong light the fact that the ills which go with this useful means of treatment are capable of being met and dealt with to the good service of cases of disease. Rest can be made to help. Rest also can hurt, and he who deals with it as a means of cure must not fail to bear in mind the modes by which we can secure the light without the shadow, the good without the harm."

---

ANALYSIS OF ONE THOUSAND CASES OF SKIN DISEASE, WITH CASES AND REMARKS ON TREATMENT. By L. Duncan Bulkley, A.M., M.D.

This is a reprint from the *American Practitioner* for May, 1875. It contains many valuable suggestions, especially in regard to treatment. We quote the following fragments from different parts of the essay:

"All the diseases noted are arranged under forty-three heads, and several of these again include a number of different forms usually recognized as separate diseases; thus acne has six varieties; eczema includes much that might be called by some impetigo and lichen; tinea includes the tinea circinata and tonsurans, or ring-worm of the body and head; also favus, parasitic sycosis, and so on.

"Considerably more than one-half of all the patients were females—five hundred and seventy-four to four hundred and twenty-four males, an excess of one hundred and fifty or fifteen per cent. of females—and in two cases the sex was not stated. But little can be learned, however, from this, as the females are more at leisure to come during the dispensary hours, and many of the diseases are more annoying to this sex than to men. But a comparatively small proportion were children, except in the case of eczema, where about one-sixth of the entire number were five years of age or under. The number of cases is too small to draw any conclusions as to the season of the year in which various diseases are wont to appear, nor can any deductions be made as to the effect of occupation in producing disease, except in certain instances. It is, however, certain to my mind, that a very large share of cases are the direct result of filth, neglect of hygienic rules, and of poor and improper food. As an instance of the effect of filth, I would cite the one hundred and forty-one cases due to animal and vegetable parasites, one-seventh of the whole number analyzed; of the neglect of hygienic considerations, the ninety-eight cases of syphilitic eruptions, or one-tenth of the whole; and of dietary errors, the one hundred and seventy-seven cases of acne, erythema, and urticaria, or one-sixth of the whole; together with the direct effect of dietary errors in causing outbreaks of eczema, furuncles, pruritus, lichen, etc.

"The diseases are further commented upon in the order of their frequency, *eczema* coming first in the list, from its frequency as well as its very great importance; and in all the statistics published it has always outnumbered any other form of cutaneous disease many-fold (with the single exception, I believe, of McCall Anderson's hospital statistics,* where scabies equals eczema in frequency), the proportion being always about the same; namely, one-third of all cases.

"It appears from these statistics that the greatest predisposition to eczema is during the first year of life, when eighteen cases were observed; and during the first five years fifty-six cases, or more than one-sixth of the whole, occurred; the next five years, five to ten, giving but twenty, or a total of seventy-six for the first decade. Wilson's statistics give but twenty-nine for the first thousand and forty-one for the second during this first decade, showing that the lower station in life has much to do in rendering the very earlier years subject to eczema. Again, the thirty years from the ages of thirty to sixty gave with Wilson one hundred and sixty-two patients with eczema in each thousand against one hundred and sixteen in our statistics. Now this period between thirty and sixty we know to be that most liable to gouty trouble, and it is quite probable that the indolent and sedative life with over-indulgence of the rich has much to do in making eczema more com-

* *Diseases of the Skin—Analysis of Eleven Thousand Cases.* London, 1872.

mon during middle life in the higher walks of English society than among the lower classes in America.

"Quite a proportion of these cases were in the young, as before stated; eighteen of the patients occurring in the first year of life, fifty-six in all being five years or under. Eczema at this period requires very careful and judicious treatment. Each case almost will require a different course; and it is well to remember that it is the *patient* which is to be treated, and not the disease. I do not order poultices to remove the crusts of infantile eczema as many do, preferring much to cause their separation by means of fatty matter. Among the poor, and sometimes among the rich, I have the head soaked in cod-liver oil (sweet-almond oil answers), or I have an ointment applied at once in a tolerably soft form; directing that the head shall not be washed at all, but as fast as the crusts fall, perhaps with slight assistance from the finger-nail, the ointment is to be re-applied; the idea being to thoroughly protect the irritated mucous layer of the skin, and to shield it from air and water. Occasionally the crusts will accumulate and adhere, and it becomes necessary to use a poultice or wash the head well with warm water and borax; but this, in my experience, is very rare.

"During the past year, I have employed very largely, tannin in ointment (one drachm to one ounce) in eczema, and like it very well. A very common treatment is to bathe first with the *liquor picis alkalinus*, diluted ten or twelve times, twice a day, and apply the tannin ointment immediately afterward. I have also used with very satisfactory results the subnitrate of bismuth in ointment (half a drachm to one ounce), and prefer it in very many instances to that of zinc, as commonly employed. I would again mention the value of the rose ointment, as an excipient, and its efficiency when the simple ointment has failed. Several cases of eczema rubrum covering quite a large part of the body of children one or two years old were seen. These cases are often most obstinate. Our best results were attained by starch and alkaline baths, and powdering the surface with subnitrate of bismuth and starch.

"Internal treatment is always required, and I believe that the largest percentage of good results was obtained by means of cod-liver oil in appropriate doses. Syrup of the iodide of iron is also invaluable in treating eczema in children.

"In adults, most of the cases of eczema were of the chronic form, very many of them being in the legs, and dependent upon varicose veins. The treatment of these is very frequently unsatisfactory, because of the continued existence of the cause, especially among the poor, who cannot give the necessary time to rest. Elastic stockings should be insisted on in eczema of the legs when the disease has recurred often or lasted long; for, although the veins may not appear to be varicose, there is often a want of tone of the capillaries, which is supplied by the stockings. We have had good results from the use of tarry preparations, and have known a moist eczema to be completely healed after a very few applications of the *liquor picis alkalinus* in full strength. A common treatment in chronic eczema is equal parts of tar and oxide of zinc ointments, with the addition of a little mercurial ointment, as the citrine, when the surface ceases to be moist.

"In one case of eczema of the scrotum, I obtained very excellent results from the repeated application, by means of a camel's-hair brush, of the compound tincture of benzoin. The man ceased attending before the thickening had entirely disappeared, and the ultimate result cannot be stated with certainty; but it is probable that the disease was cured, as the remedy was the first one tried by me, and the relief and satisfaction expressed by the patient was very great.

"Quite a large share of the cases of ordinary eczema of various parts was treated by the oxide of zinc ointment, very generally in conjunction with some internal medication, depending upon the state of the patient. Many of this class are the constant subjects of dyspepsia, and the rhubarb and soda mixture was very commonly used. I frequently add Fowler's solution to it; giving of the latter three or four drops with a teaspoonful of the former. Many of these patients require tonics, and the ammonio-citrate of iron and compound tincture of cinchona were generally used. Acute lichenous eczema I frequently treated with Startin's mixture of sulphate of magnesia, sulphate of iron, aromatic sulphuric acid, and gentian. Acetate of potassa, alone or combined, was used somewhat, and in my hands has done much for eczema.

"2. *Acne.*—Acne appears second on our list in point of frequency, and we are quite willing to accord it that place so far as the annoyance to the patient (and ofttimes the physician) is concerned. Although acne belongs rather to those of luxurious habit, it is not an uncommon affection among the poor of this city, and especially those who lead indoor lives; hence it is that by far the larger number affected are females (although it is true that, it being more a matter of vanity and not a disease affecting the welfare of the patient, males pay less attention to it.)

"The acne simplex is decidedly a disease of adolescence. The youngest persons in whom it was observed were in two girls aged fourteen; two boys aged fifteen were recorded also with this form. During the five years, from fifteen to twenty, thirty-five cases of acne applied for treatment, and twenty-nine from twenty to twenty-five years of age; thus making the total number before twenty-five years of age sixty-six, and after this period but forty-five.

"It is not easy to make out the causes of disease among this class of patients, as the time given for treating them is very limited, and the notes made are often hurried. A large share of the cases, however, depended more or less directly upon the occupation, habits, and diet of the patient. Very many were directly associated with constipation and dyspepsia, as I have elsewhere shown,* and a few doubtless with uterine derangement, as many of the symptoms were often complained of; but no uterine examination is possible in this run of practice.

"Very many of the acne patients were benefited by the internal administration of acetate of potassa

* *American Practitioner*, December, 1872.

(fifteen grains, three times daily, well diluted) on an empty stomach; but the effect of this must be followed up by tonics. Arsenic, in my experience, is of but very little use in acne. I have during the past two or three years employed quite largely the plan of treatment suggested by Gubler, of Paris—namely, the internal use of glycerine, in doses of from one to three or four teaspoonfuls three times a day, after eating—and with good results. At first I colored and flavored it, but latterly I have given the citrate of iron and quinine in it, which effects both, and assists its tonic action. This plan is especially suited to those whose skins are greasy or muddy looking, with many comedones. I have had it fail repeatedly in the rosaceous form.

"Locally, most of my patients used hot water with good results, and I have prescribed more largely than any other, a lotion containing one drachm of washed sulphur, four drachms of sulphuric ether, and three and a half ounces of alcohol, and with very generally good effect. I have rarely employed the bichloride of mercury in washes, but sometimes the well-known wash of sulphur, camphor, and water.

"3. *Syphilodermata.*—It is a sad fact that third on our list as to frequency of occurrence, come the cutaneous lesions produced by syphilis; and sadder yet, that of the ninety-eight cases of this which were recorded, seventy occurred in females to twenty-eight males. Syphilis thus gave rise to nearly ten per cent. of all the cases of skin disease. This does not include the primary sores, they seldom, if ever, appearing in this class. This percentage is much greater than that given in most other statistics; thus in McCall Anderson's ten thousand dispensary patients, but a trifle over five per cent. of the diseases were of specific origin; at the London Hospital for diseases of the skin, only about five per cent.; at St. John's Hospital for diseases of the skin, only about three per cent.; at the Boston Dispensary for diseases of the skin, the proportion was about seven per cent.; and at the Philadelphia Dispensary, about eleven per cent.

"The majority of the cases were those of the later manifestations of syphilis, the earlier or macular eruption appearing more seldom. Thirty-one were recorded as having tubercular syphilis; many others noted as having merely tertiary symptoms.

"The treatment in most of my cases has been the bichloride of mercury (one thirty-second of a grain) with from seven to fifteen grains of iodide of potassium in mixture, with tonics, thrice daily. I have combined the carbonate of ammonia with the potassium in many cases, with rather better results than from the iodide alone. Inunction was but seldom used.

"4. *Phthiriasis and Scabies.*—Animal parasitic diseases also formed nearly one-tenth of the whole number of our cases. Of the ninety-three present, fifty-seven were due to the varieties of pediculus, or louse, and have the name *phthiriasis*; and thirty-six to the acarus scabei, forming *scabies*, or itch. These diseases, preventable by cleanliness, are, I am glad to say, very much less frequent here than in Scotland; for McCall Anderson states that scabies forms fully twenty-five per cent. or one-fourth of the whole number of skin patients among the poor in Glasgow, and Milton over eighteen per cent. at the St. John's Hospital for diseases of the skin in London. It is to be hoped the proportion here will be even more lessened as intelligence and sanitary knowledge pervades the community.

"Our common method of treatment of the head-lice among the poor is, to soak the head well for twenty-four hours in kerosene oil, which destroys all the bugs and their nits; then wash the head well with soap and warm water, comb it out, and saturate it with cod-liver oil till all the sore places are healed. This treatment is very rapid and very sure, three or four days, or a week at most, serving to heal all excoriations. Or white precipitate or citrine ointment, diluted two or three times, applied from the first, will answer in many cases, and is perhaps the best in private practice. Its value is increased by having it highly scented with rosemary or some other volatile oil. For lice of the body we generally use a combination of one drachm of caustic potash and two drachms of carbolic acid in four ounces of water, using it diluted once or twice at first, directing also the clothing to be sprinkled with it. To make the cure of any of these forms permanent, the clothing must be either boiled or baked for a long time, also the bed linen, and often the bedding itself requires attention.

"5. *Psoriasis.*—The cases of psoriasis, fifty in number, were pretty evenly divided between the sexes, twenty-seven females to twenty-three males. The youngest patient was nine years of age, a boy; the oldest, seventy, also a male; and another case of a man aged sixty. Many of the cases were of great duration; one case is noted as having lasted twenty-four years in a woman aged thirty-nine; another eighteen years in a woman of thirty-seven; of course, with exacerbations and remissions; the case of the man aged seventy was recorded as psoriasis inveterata, but no duration is stated.

"Most of these patients were treated largely by local measures alone, because of the well-known obstinacy of the disease and the frequent neglect of treatment, whereby much that had been done would often be wasted. The preparations of tar, *liquor picis alkalinus*, or Hebra's compound tincture of green soap and oil of cade, were principally used, with diluted mercurial ointments. In many of the more recent cases, arsenic and alkalies were given, but no conclusions as to results can be drawn. I have obtained, however, very marked effects from the internal administration of tar and potash as combined in the *liquor picis alkalinus*, which we have so often presented to the profession * (picis liquidæ, ʒ ij; potassæ causticæ, ʒ j; aquæ, ʒ v.), giving from fifteen to thirty drops, largely diluted, on an empty stomach. It has the direct effect of diminishing the cutaneous congestion and lessening the scales. When long-continued it may disagree with the stomach, but it is a remedy of value when others fail. Three of the patients with psoriasis—two males, each aged forty, and one female, aged forty-four—had acne rosacea. No necessary connection between the two diseases is known. In none of the cases of psoriasis

* *Archives of Scientific and Practical Medicine*, February, 1873 (Brown-Sequard's).

which came under my observation, were the tongue or lips affected; all were examined, I believe. I mention this as some have intimated a connection between the lesion known as *psoriasis linguæ* and this disease."

---

ON SPASMODIC URETHRAL STRICTURE. By F. N. Otis, M.D., Clinical Professor of Genito-Urinary Diseases at the College of Physicians and Surgeons, New York. New York: G. P. Putnam's Sons, 1875.

This is a pamphlet reprinted from the *Archives of Dermatology*. The views of all the principal writers and authorities as to the nature and relative frequency of spasmodic urethral stricture are first reviewed and summed up as follows:

"In comparing the views of these recent accepted authorities, in regard to spasmodic urethral stricture, it will be observed that all agree as to its frequency, its transient character, and its easy management.

"Dittel met with a case where the pressure of the end of a catheter for fifteen minutes, against the face of a spasmodic stricture, at the membranous portion, was required before it yielded. Van Buren and Keyes have evidently had similar experiences, as they note this occasional *persistence* of the spasmodic barrier. Sir Henry Thompson inclines to ignore the existence of the spasmodic stricture, and attributes to ignorance and incapacity the arrest of an instrument in its passage into the bladder, from any cause but an organic one. In this Mr. Erichsen seems quite inclined to agree, although appreciating the possible occurrence of a spasmodic stricture which should be so persistent that it *might* be mistaken for an organic contraction.

"It is not my purpose at this time to discuss the general question of spasmodic stricture. The recent investigations of Stilling (coinciding with those of Kolliker) would seem to show conclusively that the muscular capacity of the urethral surroundings are quite sufficient to account for any amount of contraction which might be observed at any point. In his own strong language (supported by several admirable illustrations of the anatomy of the corpus spongiosum urethræ) he says:* "*the corpus spongiosum is a muscle through which the urethra runs.*" Dr. Stilling so demonstrates the muscular structure of this body that it is at once seen to be an easy matter for such a contraction of the muscular structure of the corpus spongiosum to bring a strong contracting force to bear upon any part of the urethral canal.

A number of clinical cases in illustration are given. The author says in regard to the cases:

"There are several points which coincide with the accepted characteristics of true, deep, organic stricture, and which, if not appreciated, would lead, of necessity, to an erroneous diagnosis, such as was originally made in each one of the cases reported.

"1. *Gradual* diminution of the stream of urine.

"2. *Persistent* frequency of micturition.

"3. *Persistent* resistance to the introduction of *large* instruments *in the hands of skilled surgeons.*

*B. Stilling, *Die rationelle Behandlung der Harnrohren Stricturen.* Erste Abtheilung, p. 9.

"4. Distinct grasping of small instruments, and a *gradual* toleration of instruments of increasing size, and, in this, so *perfectly* simulating the behavior of true organic stricture, that the most skilled and learned surgeons have been deceived by these conditions.

"5. The *persistence*, during a long period of years, of all symptoms which are recognized by authorities, as characteristic of *organic stricture.*

"'The grand distinguishing feature,' says Sir Henry Thompson,* 'which marks the phenomena (of spasmodic strictures), and by which they are contrasted with organic strictures, is their *transitory character.*' So says, in effect, Dr. Erichsen, Dr. Bumstead, Drs. Van Buren and Keyes, Drs. Stilling, Dittel, etc., leading teachers and authorities in such matters.

"Now, if this is *not the fact* (and that it is not the cases cited go to prove), it will be readily seen that those surgeons who differentiate organic from spasmodic strictures by what is claimed to be 'the *distinguishing feature*, viz.: the *transitory character of spasmodic stricture*,' are liable to fall into the grave error of treating a reflex urethral spasm for organic stricture. It is not at all likely that the six cases I have reported, in which this error was made (in *four* cases by *none* who did not fully understand and appreciate *all the points* which Sir Henry Thompson and Mr. Erichsen and others so explicitly lay down for guidance in such cases), I say it is not *likely* that these are *all* the cases in which such errors have occurred, or are likely to occur. They are *types of a class*, and a large one too, which will necessitate the acceptance of *other* means of diagnosis than those now in vogue, before such errors can with certainty be avoided. First of these is the necessary knowledge of the *normal calibre* of the urethra, in which symptoms of stricture are present; second, *the size and condition of the external opening*. If the measurements of these two points do not completely correspond, there is reason to believe that a reflex irritation may be present, which has the power of obscuring diagnosis. If there is a stricture at or near the meatus urinarius, acquired through a previous gonorrhœa, or of congenital origin, contact of urine with the sensitive mucous surface (which is always present behind such stricture), or contact of exploring instruments, is capable of exciting a spasm at the membranous portion of the urethra; a spasm which will often *persist* even when the patient is fully anæsthetized; and will continue up to the time that a *complete* division of the stricture is effected.

"It may, I think, be safely claimed that no reliable examination of the deeper urethra can ever be made while a *stricture*, or even an *erosion*,* is present in the anterior portion of the canal. Inferentially, then, no *treatment* of deep stricture, *per se*, should be attempted until the *complete* freedom from organic contraction of the anterior portions of the urethra is established. A long series of careful observation of the urethral calibre (by the aid of the *urethra-meter*), have conclusively demonstrated a nearly uniform relation between the size of the urethra and that of the penis in which it is located. As I have stated in other papers on this subject, that the circumference of the presenting

* Op. Cit., p. 140.

* Thompson, Op. Cit., p. 132.

penis being three inches, the normal urethral calibre will correspond to 30 or more of the French scale; if three and one-fourth, to 32 or more; if three and one-half, to 34 or more; if three and three-fourths, to 36 or more; if four, to 38 or more; if four and one-fourth, to 40 or *more*.

"When the *urethra-meter* is not available, a urethral calibre based upon these calculations may be implicitly relied upon, as not over estimated; on the contrary, it will often be found one or more millimetres below. Urethral examinations with a *bulbous sound*, corresponding in size to the normal urethral calibre, *alone* can demonstrate complete freedom from stricture in any given case. The presence of the slightest contraction at any point may be accepted as capable of producing reflex irritation, which may result in spasmodic contraction, which shall possess all the recognized characteristics of a deep organic stricture."

---

RADICAL TREATMENT OF PROSTATIC HYPERTROPHY.—Prof. Heine has cured six cases of prostatic hypertrophy with iodine injections, and now recommends the parenchymatous injection of moderately concentrated solutions of iodide of potassium; he states in *Langenbeck's Archiv*, quoted in the *New York Medical Journal*, that the operation is not severe, and can be borne by old and weak individuals, because the diminution of the hypertrophied organ takes place without suppuration. When its volume is diminished, the secondary affections of the bladder are also relieved, provided they have not attained a high degree. The operation is performed by placing the patient on his side at the edge of the bed, and introducing the oiled index-finger of the left hand into the rectum to the point where it is intended to make the injection. An exploring trocar is then introduced on the finger, the stylet having been withdrawn into the canula, and the puncture is made. The stylet is then withdrawn from the canula, which is filled with the solution in a syringe. When the canula has been filled, an air-tight syringe is attached to the canula, and the injection performed. The median line of the prostate should not be chosen, as a small artery takes its course in this location. The author's solution is: Iodidi potass., ʒ ij.; tr. iodinii, ℥ ij.; aq. destil., ℥ ij.

COMPRESSION OF THE LEFT SUBCLAVIAN ARTERY.—On the 2d inst., Mr. McGill, of Leeds, performed an interesting operation in a case of left subclavian aneurism, by cutting down upon the first part of the left subclavian artery, and compressing it for ten hours and a half by means of torsion forceps. The case had been previously treated with great temporary benefit by galvano-puncture. The details of the previous operations were published in the *Lancet* in 1874, vol. II., p. 9. After numerous galvano-punctures, the patient was able to follow her occupation of mill cook without any discomfort. For many weeks there was very slight pulsation in the aneurism, but gradually the pulsation became more forcible, and further interference was rendered necessary. Up to the time of going to press, the patient was doing well. It may be observed that the first part of the subclavian artery has only been ligatured *once*—namely, by Dr. J. K. Rodgers, of New York, in 1846.—*Lancet*, Jan. 9, 1875.

ANATOMICAL PREPARATIONS,

Including Skeletons, and the Separate Bones of the Human Subject, can be obtained at reasonable prices, of

E. H. SARGENT,
Dealer in Surgical Instruments,
CHICAGO.

